# Targeting Intracellular Pathways in Atopic Dermatitis with Small Molecule Therapeutics

**DOI:** 10.3390/cimb47080659

**Published:** 2025-08-15

**Authors:** Georgiana Nitulescu, Octavian Tudorel Olaru, Corina Andrei, George Mihai Nitulescu, Anca Zanfirescu

**Affiliations:** Faculty of Pharmacy, “Carol Davila” University of Medicine and Pharmacy, Traian Vuia 6, 020956 Bucharest, Romania; georgiana.nitulescu@umfcd.ro (G.N.); octavian.olaru@umfcd.ro (O.T.O.); corina.andrei@umfcd.ro (C.A.); anca.zanfirescu@umfcd.ro (A.Z.)

**Keywords:** Janus kinase inhibitor, phosphodiesterase 4 inhibitor, STAT6 degrader, aryl hydrocarbon receptor ligand, histamine H4 receptor antagonist, sphingosine-1-phosphate receptor modulator, type 2 inflammation targeting, targeted immunomodulator, precision dermatology, clinical trials

## Abstract

Atopic dermatitis (AD) is a chronic, relapsing inflammatory skin disorder characterized by immune dysregulation and epidermal barrier dysfunction. Advances in understanding the interplay of genetic predisposition, cytokine signaling, and environmental triggers have led to the emergence of targeted therapies. Although biologic agents such as dupilumab, tralokinumab, and lebrikizumab have revolutionized AD management, their high costs, injectable administration, and limited global accessibility highlight the need for alternative options. Small molecule therapies are gaining momentum as they target intracellular pathways central to AD pathogenesis and offer oral or topical administration routes. This review provides a comprehensive analysis of key agents including Janus kinase (JAK) inhibitors (upadacitinib, abrocitinib, baricitinib, ruxolitinib, delgocitinib), phosphodiesterase 4 (PDE4) inhibitors (crisaborole, difamilast, roflumilast, apremilast), as well as STAT6 degraders (KT621, NX3911), aryl hydrocarbon receptor modulators, histamine H4 receptor antagonists (adriforant, izuforant), and sphingosine-1-phosphate receptor modulators (etrasimod, BMS-986166). We summarize their mechanisms of action, pharmacological profiles, and pivotal clinical trial data, emphasizing their potential to address unmet therapeutic needs. Finally, we discuss safety concerns, long-term tolerability, and future directions for integrating small molecule therapies into precision treatment strategies for moderate-to-severe AD.

## 1. Introduction

Around 20–25% of people are affected by chronic, non-infectious inflammatory skin conditions. The most common include atopic dermatitis (AD), psoriasis, urticaria, lichen planus, and hidradenitis suppurativa. In many cases, an autoimmune component plays a major role [1]. AD is the most prevalent of them and is generally characterized by intense pruritus, dry skin, localized erythematous rash, recurrent eczematous lesions, but also on the patient’s family history [2]. Approximately 80% of AD cases develop during infancy or childhood, while the remaining proportion emerges later in adulthood. Globally, the prevalence of AD ranges from 2% to 10% among adults and between 2.7% and 20% in pediatric populations [3]. The disease exhibits a broad spectrum of clinical manifestations, which can complicate diagnosis. The clinical evaluation is primarily based on the characteristic morphology and distribution of skin lesions, a history of relapsing disease, and the exclusion of other dermatoses. The Hanifin–Rajka criteria remain the most widely cited and utilized [4,5].

Pathophysiologically, AD is a heterogeneous and multifactorial disease characterized by complex interactions between genetic predisposition, immune dysregulation, epidermal barrier defects, and environmental triggers. A central feature is the dysregulated type 2 helper T cell (Th2) immune response, with key cytokines such as interleukin (IL)-4, IL-13, and IL-31 driving inflammation, pruritus, and barrier dysfunction. These cytokines promote IgE production, eosinophil activation, and suppression of filaggrin (FLG) and other barrier-related proteins, perpetuating a vicious cycle of barrier impairment and allergen penetration [6]. Epidermal barrier dysfunction is further exacerbated by loss-of-function mutations in the FLG gene. FLG deficiency leads to increased transepidermal water loss, decreased natural moisturizing factors, and greater skin permeability to irritants and microbes. In addition to FLG, mutations in other epidermal differentiation complex genes (e.g., loricrin, involucrin) and tight junction proteins (e.g., claudin-1) contribute to the impaired barrier [7].

The clinical trial evaluation of new potential solutions for AD is challenging due to the multifaceted expression of the disease and subjective symptoms such as pruritus and sleep disturbance. To robustly quantify therapeutic efficacy, a series of instruments have been implemented. Among the most widely adopted are the Investigator’s Global Assessment (IGA), the Eczema Area and Severity Index (EASI), and the SCORing Atopic Dermatitis (SCORAD), each embodying distinct methodological frameworks with important implications for trial design and regulatory decision-making [8,9]. A detailed comparison of these outcome measures, including their descriptions, strengths, and limitations, is provided in Table 1.

The IGA is widely used in AD trials because of its simplicity and regulatory acceptance. It provides a quick, overall estimate of disease severity and is often the primary endpoint in pivotal studies, with success defined as a ≥2-point drop and a final score of 0 (clear) or 1 (almost clear). Its categorical nature limits sensitivity to incremental improvements that may still be clinically meaningful [10]. EASI takes a more detailed approach, scoring lesion severity and extent across four body regions and adjusting for surface area. This quantitative method reduces some subjectivity but requires time and training for consistent use, particularly in multicenter trials. A commonly reported outcome in clinical studies is EASI-75, defined as a 75% reduction from baseline in EASI score and considered a benchmark for meaningful improvement. Related thresholds, such as EASI-50 and EASI-90, are also used to reflect partial and near-complete clearance, respectively [12]. SCORAD provides a broader assessment by integrating physician-rated signs with patient-reported symptoms, including pruritus and sleep disturbance. As a composite score that incorporates both objective and subjective elements, it captures dimensions of disease burden not addressed by clinician-only measures. Nonetheless, its complexity and reliance on subjective components have restricted its use in large-scale trials, where endpoints such as IGA and EASI are more commonly favored [9].

Biologic therapies have revolutionized the treatment landscape for moderate-to-severe AD, achieving unprecedented levels of disease control and symptom relief. However, their widespread use is hampered by high production costs, parenteral administration routes, long-term safety considerations, and limited availability in low- and middle-income countries. Furthermore, the complex pathophysiology of AD, suggests that therapies targeting a single extracellular cytokine may not be sufficient for all disease phenotypes [15]. This review aims to critically assess the progress, challenges, and future prospects in the development of small targeted molecules for AD. Special attention is given to their potential to address unmet needs left by biologics, including cost, accessibility, and broader applicability across diverse patient populations.

## 2. Biologic Therapies

The introduction of biologic therapies has transformed the treatment landscape for moderate-to-severe AD, providing targeted modulation of immune pathways central to disease pathogenesis.

Dupilumab was the first biologic approved for AD by both the U.S. Food and Drug Administration (FDA) and the European Medicines Agency (EMA) in 2017, and it remains a cornerstone of targeted therapy. As a fully human monoclonal antibody (mAb) against the interleukin-4 receptor alpha subunit (IL-4Rα), it inhibits signaling of both IL-4 and IL-13, the principal drivers of Th2 inflammation. Dupilumab’s safety profile is favorable, though conjunctivitis and injection site reactions are relatively common adverse events [16,17]. Tralokinumab and lebrikizumab are mAbs that target IL-13 directly and have recently been approved as additional treatment options for adolescents and adults (≥12 years) with moderate-to-severe AD inadequately controlled by topical therapies (tralokinumab: EMA 2021; FDA 2021; lebrikizumab: FDA 2023; EMA 2023). Adverse effects are generally mild, with conjunctivitis and upper respiratory infections reported most frequently. Lebrikizumab acts by preventing the formation of the IL-13Rα1/IL-4Rα heterodimer, thereby blocking downstream signal transducer and activator of transcription (STAT) 6 activation. It is approved for patients aged 12 years and older with moderate-to-severe AD not adequately controlled with topical therapies (FDA 2024, EMA 2023). Its safety profile is comparable to tralokinumab, with conjunctivitis reported as a common, though manageable, adverse event [18,19]. Nemolizumab is a humanized mAb that selectively binds to IL-31 receptor alpha (IL-31RA). It is approved FDA (2024) for adolescents and adults aged 12 years and older with moderate-to-severe AD [20,21].

Several mAbs targeting alternative inflammatory pathways are under investigation for AD. Afimkibart targets the tumor necrosis factor-like cytokine 1A (TL1A) and is currently in phase II trials, aiming to modulate broad inflammatory cascades [22]. Bermekimab blocks IL-1α and is also in phase II studies for its potential to suppress keratinocyte-driven inflammation [23]. Tozorakimab acts on IL-33, an epithelial alarmin implicated in AD pathogenesis, while ucenprubart, a CD200R1 (Cluster of Differentiation 200 Receptor 1) agonist, explores immune checkpoint activation to dampen skin inflammation. Together, these agents represent promising strategies beyond type 2 cytokine inhibition [22,24].

The OX40–OX40L axis, involving OX40 (TNFRSF4/CD134) on activated T cells and its ligand OX40L (TNFSF4/CD252) on antigen-presenting cells, is a critical co-stimulatory pathway sustaining effector and memory T-cell responses in AD. Rocatinlimab (AMG 451/KHK4083) is a fully human mAb against OX40. Amlitelimab (KY1005) targets OX40L and blocks T-cell/APC signaling. Telazorlimab (ISB 830/GBR 830), administered subcutaneously, is another anti-OX40 mAb showing promising early-phase results. All three agents demonstrated favorable safety profiles and suggest the possibility of long-lasting immunological reset in AD [25,26].

While biologics targeting extracellular cytokines such as IL-4 and IL-13 have demonstrated substantial clinical benefit, there is increasing interest in small molecule therapies capable of modulating intracellular signaling pathways integral to AD pathogenesis. These agents, which are often orally or topically administered, offer advantages in terms of pharmacokinetics and accessibility and may provide alternative therapeutic options for patients. Unlike monoclonal antibodies, which are limited to targeting extracellular cytokines or receptors, small molecules can penetrate cell membranes to modulate key signaling intermediates within inflammatory pathways. This capability allows them to simultaneously influence multiple cytokine signals converging on shared intracellular nodes, potentially leading to broader anti-inflammatory effects. The identification of intracellular targets for small molecule therapeutics represents a significant advance in the management of AD. Inhibitors of Janus kinases (JAKs) and Phosphodiesterase 4 (PDE4) have already established clinical utility, while emerging strategies targeting STAT6 and other intracellular proteins hold the potential to further expand treatment options. Ongoing clinical trials will clarify the efficacy, safety, and long-term tolerability of these agents relative to established biologics [27,28].

## 3. Janus Kinases Inhibitors

### 3.1. Background

Janus kinases (JAKs) comprise four intracellular, non-receptor tyrosine kinases: JAK1, JAK2, JAK3, and tyrosine kinase 2 (TYK2)**.** They mediate signaling from type I and type II cytokine receptors, which lack intrinsic enzymatic activity. These receptors include those for interleukins (e.g., IL-2, IL-4, IL-6, IL-7, IL-12, IL-23), interferons (IFN-α, IFN-β, IFN-γ), and colony-stimulating factors such as GM-CSF (Granulocyte-Macrophage Colony-Stimulating Factor). Upon ligand binding, JAKs become activated and phosphorylate downstream signal transducers and activators of transcription (STATs). The phosphorylated STATs dimerize and translocate to the nucleus, where they regulate the expression of genes involved in cell proliferation, differentiation, survival, and immune function [29].

**Figure 1:**
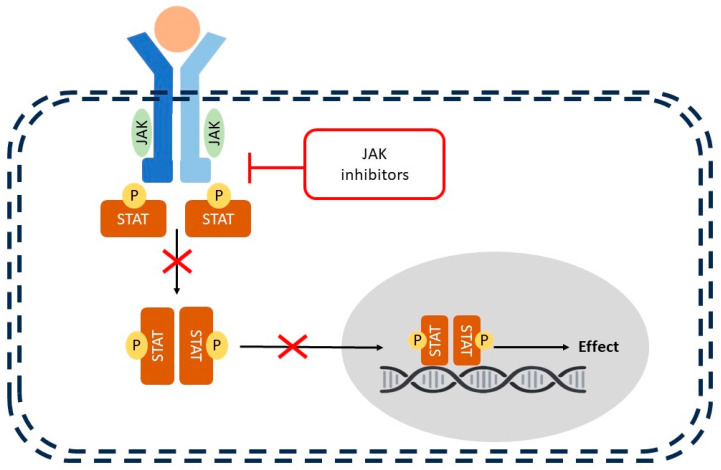
Simplified schematic of the JAK-STAT signaling pathway, representing the general mechanism of action of Janus kinase (JAK) inhibitors in atopic dermatitis (AD).

Figure 1 illustrates an overview of the JAK-STAT pathway and its key components. Cytokine binding activates JAKs, which phosphorylate STAT proteins, enabling their dimerization and nuclear translocation to regulate gene transcription. JAK inhibitors block this phosphorylation step, thereby preventing STAT activation and downstream gene expression.

### 3.2. Representatives

JAK inhibitors (JAKi) constitute a class of targeted small molecules that interrupt cytokine signaling implicated in the pathogenesis AD. Clinical trials have demonstrated rapid reductions in pruritus and objective disease scores, making these agents an important alternative to other therapies. However, their use is associated with several issues, including an increased risk of upper respiratory infections, herpes zoster, opportunistic infections, venous thromboembolism, and major adverse cardiovascular events. These findings have prompted regulatory authorities to mandate boxed warnings and recommend baseline and periodic laboratory assessments. Due to their risk profile, JAK inhibitors are generally reserved for patients with moderate-to-severe AD [30,31].

**Figure 2:**
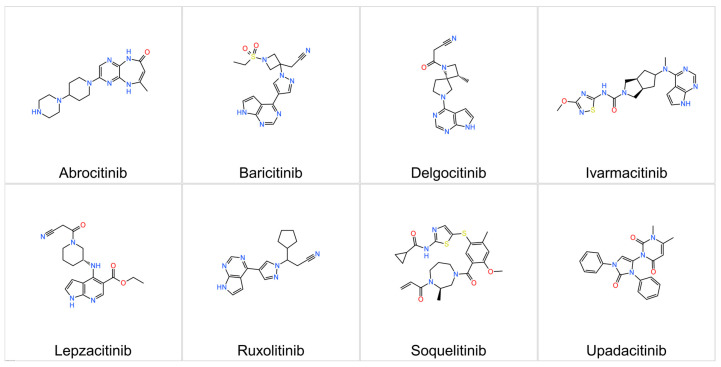
Chemical structures of representative JAK family inhibitors that are used or in clinical trials for atopic dermatitis (AD).

Here we review the JAKi currently approved or investigated for AD, highlighting their diversity in selectivity and formulation. Oral selective JAK1 inhibitors such as upadacitinib, abrocitinib, and ivarmacitinib, and the dual JAK1/JAK2 inhibitor baricitinib, provide systemic control for moderate-to-severe AD. Gusacitinib represents a pan-JAK/SYK inhibitor, while soquelitinib and ATI-2138 extend their inhibition spectrum by inhibiting interleukin-2-inducible T-cell kinase (ITK) and JAK3. Topical therapies such as ruxolitinib (JAK1/JAK2) and delgocitinib (pan-JAK) offer localized action with minimal systemic exposure. Selective inhibitors may reduce off-target effects, while pan-JAK inhibitors could achieve broader cytokine suppression. Given the overlapping immunopathogenesis of AD with other inflammatory dermatoses, we suggest that JAKi effective in related conditions (e.g., prurigo nodularis, chronic hand eczema, hidradenitis suppurativa) could represent future candidates for AD therapy. Nonetheless, careful long-term safety evaluation will be essential as this class continues to expand. Figure 2 illustrates the chemical diversity of representative JAK inhibitors discussed here.

#### 3.2.1. Upadacitinib

Upadacitinib is a selective JAK1 inhibitor administered orally, characterized by high target specificity and potent immunomodulatory properties. Enzymatic profiling showed that upadacitinib inhibits JAK1 with an IC_50_ of 43 nM and exhibits approximately 2.8-fold, 53-fold, and 109-fold reduced potency against JAK2, JAK3, and TYK2, respectively [32]. It has been approved by FDA (2022) and EMA (2021) for the treatment of moderate-to-severe AD in adults and adolescents aged 12 years and older. By preferentially inhibiting JAK1, it disrupts signaling pathways mediated by key pro-inflammatory cytokines implicated in AD pathogenesis, such as IL-4, IL-13, IL-22, IL-31, and IFN-γ. This targeted mechanism of action contributes to the reduction of cutaneous inflammation, pruritus, and restoration of epidermal barrier function [33].

Pharmacodynamic studies demonstrate that upadacitinib achieves nanomolar-range inhibition of JAK1, with substantially diminished activity against other JAK family members, thereby minimizing off-target effects commonly associated with less selective JAK inhibitors. The therapeutic efficacy and safety profile of upadacitinib have been validated in multiple pivotal phase 3 clinical trials, including Measure Up 1, Measure Up 2, and AD Up. In these studies, once-daily dosing at 15 mg and 30 mg was associated with significant improvements in clinical endpoints, including the EASI, IGA, and pruritus numeric rating scale (NRS), relative to placebo. Notably, some patients experienced a rapid onset of itch relief within two days of treatment initiation [34,35].

The safety profile of upadacitinib is consistent with its pharmacological class. The most frequently observed adverse events include acne, nasopharyngitis, and headache. There is a dose-dependent increase in the incidence of herpes zoster infections and elevations in creatine phosphokinase (CPK) levels. Although rare, serious adverse events such as venous thromboembolism and severe infections have been reported, predominantly at the higher 30 mg dose. As such, long-term safety monitoring is recommended, particularly in individuals with predisposing risk factors [36]. Upadacitinib is now approved in several regions, including the United States, European Union, and Japan, as a systemic treatment option for patients with moderate-to-severe AD who are candidates for oral therapy [37].

#### 3.2.2. Abrocitinib

Abrocitinib exhibits high potency for JAK1 (IC_50_ ≈ 29 nM) and shows markedly reduced activity against other JAK isoforms, including JAK2 (IC_50_ ≈ 803 nM), TYK2 (≈1.3 µM), and JAK3 (>10 µM), supporting its classification as a JAK1-preferential inhibitor [38]. Structurally, abrocitinib features a pyrrolo[2,3-d]pyrimidine scaffold—a common motif among JAK inhibitors. This configuration facilitates high-affinity binding to the ATP-binding site of JAK1, with crystallographic and docking studies confirming a type I inhibition profile. Abrocitinib stabilizes the kinase in its active DFG-in conformation, and engages in multiple key interactions, including hydrogen bonding with hinge-region residues such as Glu957 and Leu959 [39].

Abrocitinib was approved in 2022 by the FDA for the treatment of moderate-to-severe AD in adults and adolescents aged 12 years and older who are candidates for systemic therapy. It is administered orally, with once-daily dosing of 100 mg or 200 mg. The drug demonstrates rapid absorption, reaching peak plasma concentrations within 1 h, and is metabolized primarily via CYP2C19 and CYP2C9. Its pharmacokinetic profile supports convenient once-daily use, and its active metabolites contribute to overall therapeutic exposure [40].

Clinically, abrocitinib has demonstrated robust efficacy across several phase 3 trials. In the JADE MONO and JADE COMPARE studies, patients receiving abrocitinib showed significant improvements in both objective and subjective measures of disease, including EASI-75 response and rapid relief from pruritus, often within the first 48 h. Its mechanism targets JAK1-dependent cytokine signaling, thereby modulating key inflammatory mediators implicated in AD pathogenesis, such as IL-4, IL-13, IL-22, IL-31, and IFN-γ [41,42].

The most common adverse effects observed with abrocitinib include nausea, headache, and acne, most of which are mild to moderate in severity. Laboratory monitoring may reveal transient decreases in platelet counts and increases in lipid levels, both of which are dose-dependent. There is also an increased incidence of herpes simplex and herpes zoster infections, consistent with the immunomodulatory effects of JAK inhibition. Serious adverse events, including venous thromboembolism and major cardiovascular incidents, have occasionally been reported, particularly at the 200 mg dose. This highlights the need for careful evaluation of patient-specific risk factors before initiating treatment [41].

With its oral formulation, rapid onset of action, and targeted cytokine inhibition, abrocitinib represents a valuable systemic therapy for patients with refractory AD. The favorable balance between efficacy and safety, especially at the 100 mg dose, makes it a strong alternative for patients who have not responded adequately to biologics or topical treatments [43].

#### 3.2.3. Baricitinib

Baricitinib is a selective JAK1/JAK2 inhibitor developed for the treatment of moderate-to-severe AD. It demonstrates high potency for JAK1 and JAK2 (IC_50_ ≈ 5.9 and 5.7 nM, respectively) and substantially lower activity against JAK3 (IC_50_ ≈ 560 nM) and TYK2 (≈53 nM), with negligible inhibition of unrelated kinases such as Chk2 and c-Met (IC_50_ > 1000 nM) [44]. Structurally, baricitinib belongs to the class of pyrrolo[2,3-d]pyrimidine derivatives, enabling selective ATP-competitive inhibition of JAK1 and JAK2. Crystallographic studies support a type I binding mode, stabilizing the active DFG-in conformation of the kinase domain and forming key interactions with hinge region residues [45,46].

Baricitinib was approved in 2021 by the FDA for the treatment of moderate-to-severe AD in adults who are candidates for systemic therapy. It is administered orally once daily, at doses of 2 mg or 4 mg. The drug is rapidly absorbed, reaching peak plasma concentrations approximately 1 h post-dose, with an absolute bioavailability of 79%. Metabolism via CYP3A4 accounts for less than 10% of its clearance, as elimination occurs primarily through renal excretion of the unchanged drug. This pharmacokinetic profile supports steady systemic exposure with minimal accumulation, allowing convenient once-daily dosing [47].

Clinically, baricitinib has shown efficacy across multiple phase 3 trials (BREEZE-AD program), both as monotherapy and in combination with topical corticosteroids. Patients treated with baricitinib achieved significant improvements in disease severity measures such as EASI-75 and vIGA-AD scores, as well as rapid relief from pruritus and sleep disturbance. The 4 mg dose offered greater efficacy, with responses observed as early as week 1 for some endpoints. Mechanistically, baricitinib inhibits JAK1/JAK2-dependent cytokine signaling, modulating inflammatory pathways driven by IL-4, IL-13, IL-22, IL-31, and IFN-γ, as well as enhancing skin barrier function through upregulation of FLG expression in keratinocytes [48,49].

In a network meta-analysis by Wan et al., all three oral JAK inhibitors demonstrated superior efficacy compared to placebo in moderate-to-severe AD. Upadacitinib, especially at 30 mg daily, consistently showed the highest efficacy across all regimens. This was followed by the lower dose of 15 mg, which was similar to abrocitinib 200 mg. Baricitinib (1, 2, and 4 mg) was the least potent in terms of efficacy [50].

The most commonly reported adverse effects of baricitinib include upper respiratory tract infections, headache, and nausea, most of which are mild or moderate in severity. Laboratory monitoring may reveal transient reductions in neutrophil counts and increases in lymphocyte counts, both typically resolving within 24 h. As with other JAK inhibitors, there is an observed risk of herpes simplex and herpes zoster reactivation. Rare but serious events such as venous thromboembolism (VTE) and major cardiovascular events have also been reported, necessitating careful patient selection and monitoring during treatment [51,52].

With its oral formulation, dual JAK1/JAK2 inhibition, and favorable efficacy profile, baricitinib represents an important systemic option for patients with refractory AD. The balance of clinical benefit and safety, particularly at the 2 mg dose, offers a viable alternative for those who do not achieve sufficient control with topical therapies or biologics [30].

#### 3.2.4. Ruxolitinib

Ruxolitinib is a potent and selective inhibitor of Janus kinases JAK1 and JAK2, exhibiting nanomolar inhibitory activity (IC_50_ ≈ 3 nM) and substantially reduced affinity for JAK3 (IC_50_ ≈ 430 nM). Structurally, it incorporates a pyrazole moiety fused to a pyrrolo[2,3-d]pyrimidine core, a configuration that underpins its high-affinity engagement with the ATP-binding pocket of JAK kinases [53]. Structural and computational studies delineate ruxolitinib as a type I inhibitor, favoring stabilization of the kinase in its catalytically active DFG-in conformation. Key molecular interactions include hydrogen bonding of the pyrrolopyrimidine scaffold with residues within the hinge region of JAK1 and JAK2, critical for its inhibitory efficacy [54].

In 2022, ruxolitinib received regulatory approval as a 1.5% topical cream for managing mild-to-moderate AD. By attenuating JAK1/JAK2-dependent cytokine signaling pathways—particularly those mediated by IL-4, IL-13, and IFN-γ—the drug effectively reduces inflammation, pruritus, and cutaneous lesion severity. The topical formulation ensures localized pharmacological action, thereby limiting systemic bioavailability and supporting a favorable safety profile [55]. Adverse effects are typically mild and localized to the application site, manifesting as transient burning, stinging, or erythema. Occasional reports of herpes simplex reactivation and superficial skin infections have also been documented [56].

#### 3.2.5. Delgocitinib

Delgocitinib (JTE-052) is a pan-JAK inhibitor developed for the treatment of AD, primarily as a topical therapy for mild-to-moderate disease. It exhibits potent inhibition across all JAK isoforms, with reported IC_50_ values of approximately 2.8 nM for JAK1, 2.6 nM for JAK2, 12.5 nM for JAK3, and 57.8 nM for TYK2. Structurally, delgocitinib contains a pyrrolo[2,3-d]pyrimidine scaffold, a structural motif common to many JAK inhibitors [57]. By broadly blocking cytokine-driven JAK-STAT signaling, it suppresses IFN-γ, IL-4, IL-13, IL-17A, IL-22 as demonstrated in vitro in T cells and mast cells from healthy donors. In murine AD models, delgocitinib mitigated STAT3-mediated barrier dysfunction and STAT6-driven inflammation via inhibition of IL-4/IL-13 signaling [58].

In two Phase 3 trials (QBA4-1 and QBA4-2), delgocitinib 0.5% ointment significantly improved mEASI scores versus vehicle at 4 weeks (−44.3% vs. +1.7%; *p* < 0.001), with sustained efficacy and a favorable safety profile over 52 weeks. The most common adverse events were nasopharyngitis, eczema, and headache [59]. Pharmacokinetic studies demonstrate minimal systemic absorption, with plasma levels remaining well below thresholds associated with systemic JAK inhibition [60].

Delgocitinib was first approved in Japan in 2020 as the world’s first topical JAK inhibitor for patients aged ≥16 years, while a 0.25% formulation was later approved for children aged 2 to <16 years with AD [61]. EMA approved in 2023 delgocitinib as 2% cream formulation for adults with moderate-to-severe chronic hand eczema who are unresponsive to or unsuitable for topical corticosteroids [62].

#### 3.2.6. Ivarmacitinib

Ivarmacitinib exhibits high selectivity for JAK1, with an IC_50_ of 0.1 nM. Its inhibitory potency is approximately 9-fold lower for JAK2 (IC_50_ 0.9 nM), 77-fold lower for JAK3 (IC_50_ 7.7 nM), and 420-fold lower for TYK2 (IC_50_ 42 nM) [63].

In the phase 3 clinical trial QUARTZ3 (NCT04875169), once-daily administration of ivarmacitinib resulted in significant improvements in disease severity and was associated with a favorable benefit–risk profile in adolescents and adults with moderate-to-severe AD. The patients treated with 4 mg or 8 mg achieved higher rates of skin clearance and symptom reduction compared to placebo at 16 weeks. Up to 42% reached a clear or almost clear status on the IGA, and 66% achieved at least a 75% improvement in EASI75. These improvements were sustained over a 52-week treatment period. Common side effects observed in AD trials included upper respiratory infections, increased CPK levels, and folliculitis [64].

Approved in China in April 2025, it is indicated for adults with moderate-to-severe AD who have not responded adequately or are intolerant to topical therapies or other systemic treatments [63].

#### 3.2.7. Lepzacitinib

Lepzacitinib, also known as ATI-1777, is a topical JAK inhibitor. Structurally, it is an ethyl ester derivative engineered to deliver potent JAK1 and JAK3 inhibition within the skin while minimizing systemic exposure. Once absorbed, it is rapidly hydrolyzed to its carboxylic acid metabolite, CDD-1913, which displays reduce JAK activity. This metabolic inactivation reduces the risk of systemic reactions. Enzymatic assays show that lepzacitinib inhibits JAK1 and JAK3 with low nanomolar potency, while its activity against JAK2 and TYK2 is lower [65].

In a Phase 2a clinical trial, adults with moderate-to-severe AD applied lepzacitinib formulations (0.5%, 1.0%, and 2.0% *w*/*w*) twice daily for four weeks. The patients treated with the 2% solution achieved a 75% reduction in modified EASI scores, compared to a 41% reduction in the vehicle group. Significantly more patients in the active treatment arm reached the EASI-50 and EASI-75 endpoints, with improvements observed as early as day 8. Systemic drug levels remained minimal, proving the effectiveness of the soft-drug strategy. The treatment was well tolerated, with adverse events comparable to vehicle and no serious drug-related reactions [65].

#### 3.2.8. Gusacitinib

Gusacitinib (also known as ASN002) is an investigational oral dual JAK/SYK inhibitor. It inhibits SYK at around 5 nM and JAK1/2/3 and TYK2 in the 4–46 nM range. It has demonstrated rapid, meaningful improvements in moderate-to-severe AD Phase Ib/II trials, and a long-term safety study (NCT03654755) [66,67].

#### 3.2.9. Soquelitinib

Soquelitinib is a covalent inhibitor of interleukin-2-inducible T-cell kinase (ITK) engineered to bind selectively to Cys-442, demonstrating over 100-fold specificity versus related TEC kinases such as RLK (Resting Lymphocyte Kinase). In vitro, it preferentially suppresses Th2 cytokines (IL-4, IL-5, IL-13) while sparing Th1 cytokines [68]. The phase 1 trial NCT06345404 evaluates soquelitinib in adults with moderate-to-severe AD refractory to standard treatments. In this randomized, double-blind, placebo-controlled study, 64 participants receive escalating doses of soquelitinib or placebo for 28 days. Interim data show the 200 mg BID dose reduced EASI scores by 64.8% at Day 28 versus 34.4% for placebo, with no dose-limiting toxicities and a favorable safety profile, supporting its potential as a novel oral therapy for AD [69].

#### 3.2.10. Jaktinib

Jaktinib (gecacitinib), a deuterated analogue of momelotinib, is a pan-JAK inhibitor. Originally developed for myelofibrosis, it has since been investigated for moderate-to-severe AD. In a Phase 2 randomized, double-blind, placebo-controlled trial (NCT04539639), the 50 mg twice-daily dose achieved an EASI-50 response in 80.9% of patients at week 12, versus 54.5% with placebo. Secondary outcomes, including EASI-75 and IGA scores, showed consistent improvement. The treatment was generally well tolerated, with adverse events mostly mild to moderate and no serious safety signals observed [70].

#### 3.2.11. ATI-2138

ATI-2138 is a first-in-class covalent inhibitor that irreversibly targets both ITK and JAK3, key kinases in T cell receptor and cytokine signaling. By suppressing these pathways, ATI-2138 has the potential to treat Th2-driven atopic and allergic diseases such as AD. Phase I trials in healthy volunteers demonstrated good tolerability up to 80 mg, linear pharmacokinetics, and dose-dependent inhibition of IL-2 and IFNγ, confirming effective JAK3 and ITK pathway blockade [71].

### 3.3. Comparative Summary of JAK Inhibitors in AD

To facilitate direct comparison of these agents, Table 2 summarizes key characteristics of the major oral and topical JAKi currently approved for AD.

## 4. STAT6 Directed Therapies

### 4.1. Background

STAT6 is a central transcription factor in IL-4 and IL-13 signaling, driving Th2 immune responses implicated in allergic diseases, fibrosis, and certain cancers. Activation begins when IL-4/IL-13 binding induces receptor phosphorylation by JAKs, creating phosphotyrosine sites for STAT6’s Src homology 2 (SH2) domain. STAT6 is then phosphorylated by JAKs, dimerizes via SH2–pY interactions, translocates to the nucleus, and initiates transcription of pro-inflammatory genes. Traditional strategies, like JAK inhibitors, lack specificity for STAT6 and risk off-target effects by broadly suppressing JAK-STAT pathways. Targeting STAT6 directly has been challenging due to its shallow SH2 pocket, poor cell permeability of phosphotyrosine mimetics, and high similarity to other STAT family members [72]. Given the central role of IL-4/IL-13 signaling in AD pathogenesis, targeting STAT6 offers a promising alternative to monoclonal antibodies by potentially providing oral, systemically active therapies [73].

STAT6 has recently become accessible to pharmacological intervention through the advent of targeted protein degradation. This strategy, exemplified by proteolysis-targeting chimeras (PROTACs), does not rely on inhibiting enzymatic activity but instead facilitates the elimination of the target protein from the cellular environment. By tethering STAT6 to E3 ubiquitin ligases, PROTACs promote ubiquitination and subsequent proteasomal degradation, thus achieving functional silencing of this key transcription factor [74].

It is important to note that KT 621, REX 8756, NX 3911, and similar STAT6-targeted therapies are still in the very early stages of development. Their success in AD will depend on confirming efficacy and safety in clinical trials.

### 4.2. KT-621

The ongoing Phase 1 clinical trial (NCT06673667) evaluates KT-621, an oral STAT6 degrader, in healthy adults aged 19–55. This first-in-human, randomized, double-blind, placebo-controlled study to assess safety, tolerability, pharmacokinetics, and pharmacodynamics. Early data indicate that KT-621 achieves over 90% STAT6 degradation in blood at doses above 1.5 mg, with complete degradation in blood and skin observed at doses exceeding 50 mg, and demonstrates good tolerability without serious adverse events. These results support further clinical development, with Phase 1b trials already underway in AD and Phase 2b studies planned for asthma and dermatitis in late 2025 [75].

### 4.3. REX-8756

REX-8756 is a first-in-class oral, reversible, and highly selective STAT6 inhibitor designed to block IL-4 and IL-13 signaling by targeting the SH2 domain of STAT6. Preclinical studies demonstrate that REX-8756 achieves complete and durable inhibition of STAT6 phosphorylation, leading to suppression of downstream inflammatory gene expression. In animal models of asthma, AD, and acute lung inflammation, the compound reduced key type 2 inflammatory biomarkers and showed therapeutic efficacy comparable to anti–IL-4/IL-13 biologics. REX-8756 does not degrade STAT6, as KT-621, but directly prevents its activation. Favorable tolerability and pharmacokinetic profiles in preclinical testing support its advancement toward clinical evaluation for AD [76].

### 4.4. NX-3911

NX-3911 is a promising oral STAT6 degrader advancing toward clinical development. In preclinical studies, it produced rapid, potent, and selective degradation of STAT6 in blood and skin tissues, resulting in complete suppression of IL-4/IL-13–driven signaling pathways. It demonstrated therapeutic efficacy in animal models of AD, asthma, and other type 2–mediated diseases. If clinical trials confirm the preclinical data, NX-3911 could provide a more convenient alternative to injectable biologics targeting IL-4/IL-13 [77].

## 5. Phosphodiesterase 4 Inhibitors

### 5.1. Background

Phosphodiesterase 4 (PDE4) enzymatically breaks down cyclic adenosine monophosphate (cAMP), a critical signaling molecule involved in the regulation of multiple physiological processes. PDE4 is found in immune and inflammatory cells such as basophils, mast cells, eosinophils, B and T lymphocytes, monocytes, macrophages, neutrophils and endothelial cells, playing a regulatory role in immune and inflammatory responses [78]. There are four subtypes of PDE4 enzymes (PDE4A-D), located on different chromosomes. These subtypes can express multiple proteins, leading to at least 25 distinct isoforms, each with varying distribution and expression in cellular compartments. PDE4 enzymes exist in three sizes: long, short, and super-short. The long form contains two upstream conserved regions (UCR1 and UCR2), while the short form has only UCR2, and the super-short form has a truncated UCR2. All PDE4 enzymes have a catalytic domain at the C-terminus, essential for their function. The enzyme’s active site includes pockets for cAMP interaction and inhibitor binding. However, the high sequence similarity among PDE4 isoforms complicates the development of isoform-specific inhibitors [79].

### 5.2. Mechanism of Action

PDE4 activity is increased in patients with AD [3]. This leads to decreased cAMP levels, which in turn activates the nuclear factor (NF)-kB pathway and heightens the production of pro-inflammatory cytokines such as interleukin (IL)-4 and IL-13, as well as of prostaglandin E2, suggesting that inhibition of PDE4 may decrease the inflammatory processes associated with AD [80].

Conversely, inhibition of PDE4 results in elevated cAMP levels. cAMP is a crucial intracellular second messenger involved in numerous signaling pathways across different cell types. Its increase typically activates protein kinase A, initiating a cascade of phosphorylation events on target proteins [81]. cAMP modulates immune and inflammatory responses, particularly in skin cells like melanocytes, keratinocytes, and fibroblasts, which are relevant to inflammatory skin conditions. cAMP regulates immune cell functions, inhibiting the production of pro-inflammatory mediators like TNF-α, IFN-β, IFN-γ, IL-12, and LTB4, while promoting anti-inflammatory mediators such as IL-10 [82,83]. T-cells, central to the pathogenesis of these skin disorders, are highly influenced by changes in cAMP levels, highlighting the molecule’s significance in controlling inflammation. Therefore, the reduction of cAMP suppresses the inflammatory cascade associated with AD, thereby reducing symptoms like itching and redness [27].

**Figure 3:**
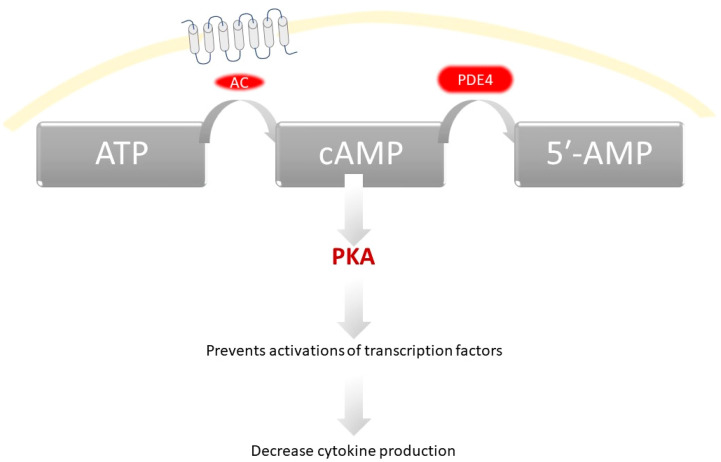
Simplified schematic of the cAMP signaling pathway, representing the general mechanism of action of phosphodiesterase-4 (PDE4) inhibitors in atopic dermatitis (AD).

Figure 3 illustrates an overview of the cAMP signaling pathway and its key components. PDE4 inhibitors block the enzymatic degradation of cAMP, thereby sustaining PKA activation and shifting immune responses toward an anti-inflammatory profile, ultimately reducing cytokine release and skin inflammation.

### 5.3. Representatives

**Figure 4:**
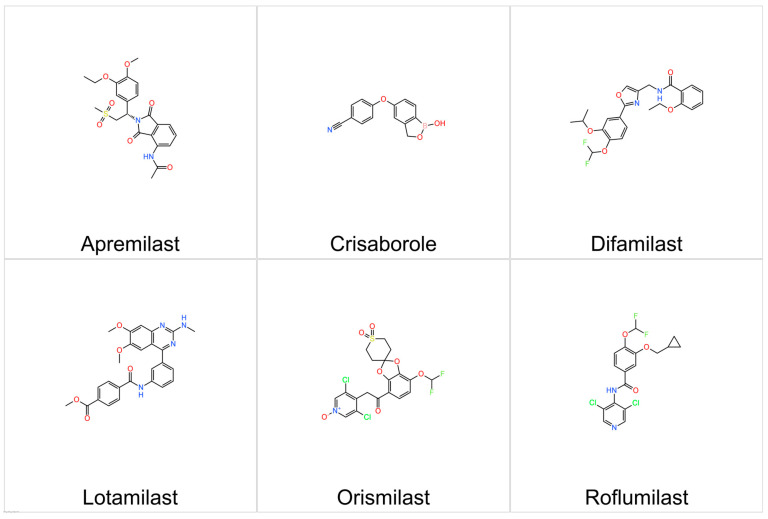
Chemical structures of representative PDE4 inhibitors that are used or in clinical trials for atopic dermatitis (AD).

Over the past decade, several PDE4 inhibitors have gained regulatory approval for diverse inflammatory and autoimmune disorders. Oral formulations, including apremilast (approved for plaque psoriasis, psoriatic arthritis, and Behçet’s disease) and roflumilast (for severe chronic obstructive pulmonary disease), have demonstrated systemic immunomodulatory efficacy. More recently, topical PDE4 inhibitors such as crisaborole and difamilast have expanded therapeutic options for mild-to-moderate AD, while topical roflumilast has been approved for plaque psoriasis and seborrheic dermatitis. These agents highlight the therapeutic versatility of PDE4 inhibition across dermatological and respiratory [84,85,86]. Figure 4 illustrates the chemical structures of representative PDE4 inhibitors discussed here.

#### 5.3.1. Crisaborole

Crisaborole is a boron-based PDE4 inhibitor that achieves selectivity through distinctive structural features. Its benzoxaborole scaffold facilitates both hydrophobic and polar interactions within the PDE4 active site, while the boron atom, acting as a Lewis acid, coordinates with zinc and magnesium ions to form a stable tetrahedral boron-oxygen complex. This coordination disrupts cAMP degradation and enhances binding affinity, contributing to selective PDE4 inhibition and minimizing off-target activity. While these attributes suggest potential advantages over traditional inhibitors like roflumilast, the clinical significance of these benefits remains to be fully established [87].

Crisaborole was the first PDE4 inhibitor approved for AD, receiving FDA approval in 2016 for patients aged ≥2 years, with the indication expanded in 2020 to include children ≥3 months. EMA approved its use in 2020 [88]. Crisaborole is available as a 2% ointment for the topical treatment of mild-to-moderate AD in adults and children aged three months and older [89]. It exhibits excellent anti-inflammatory activity both in vitro and in vivo, with animal toxicity studies indicating a wide safety margin for systemic and topical use [90]. Clinical trials confirmed the efficacy of crisaborole for treating mild-to-moderate AD, confirming its good safety profile. One clinical trial analyzed the efficacy of crisaborole in patients from different racial and ethnic groups, showing significant improvements in disease severity and quality of life across all groups. The most common adverse event was application site pain. No serious adverse events were reported [91]. Another study on skin biomarkers showed that crisaborole rapidly reduced inflammation, improved skin barrier function, and normalized the expression of key inflammatory biomarkers in AD lesions [92]. In infants aged 3 to 24 months with mild-to-moderate AD, crisaborole was effective and well-tolerated, with no significant systemic exposure [93]. Crisaborole offers a safe and effective option for treating mild-to-moderate AD, with a favorable safety profile, rapid symptom relief, and suitability for use in pediatric patients [94].

#### 5.3.2. Roflumilast

Roflumilast is a highly potent and selective PDE4B/D inhibitor, active at sub-nanomolar concentrations, and effectively blocks type 2 cytokine-mediated inflammation. It binds to the catalytic sites of PDE4B and PDE4D in a manner that structurally mimics key aspects of cAMP recognition. The 2,6-dichloropyridyl moiety inserts into the M pocket, forming a hydrogen bond via its nitrogen atom with a Mg^2+^-coordinated water molecule. Meanwhile, the difluoromethoxy and cyclopropylmethoxy moieties extend into the Q1 and Q2 sub-pockets. The dialkoxyphenyl group of roflumilast is sandwiched within the hydrophobic clamp, in a manner analogous to the adenine fragment of cAMP [95]. This binding mode explains the high inhibitory capacity of the compound.

Topical administration of roflumilast is preferred over oral dosing, as it achieves significantly higher skin concentrations while maintaining markedly lower systemic exposure, thereby minimizing adverse effects. A reservoir effect in the stratum corneum supports sustained release (t½ ~ 4 days) [96]. The clinical trials demonstrated good outcomes in AD. Roflumilast cream (0.15% and 0.05%) was applied once daily for four weeks in patients with mild-to-moderate AD. Both concentrations resulted in significant improvements in the EASI compared to the vehicle group. Additionally, a higher proportion of patients achieved a “clear” or “almost clear” score on the IGA for AD. The treatment was well tolerated, with mild treatment-related adverse effects like rash and application site pain. Only one patient discontinued the study due to adverse events, suggesting that roflumilast is a safe and effective nonsteroidal option for managing AD [97].

Two phase 3 trials, INTEGUMENT-1 and INTEGUMENT-2, assessed the efficacy of roflumilast cream 0.15%, applied once daily for four weeks in patients aged 6 years and older with mild-to-moderate AD. The primary endpoint, clear/almost clear skin and a ≥2-grade improvement, was significantly higher in the roflumilast group (31.3%) compared to the vehicle group (14.1%). Symptoms like pruritus, sleep quality, quality of life also significantly favored roflumilast, while safety and tolerability of the treatment were favorable [98]. The INTEGUMENT-PED trial extended the study to children aged 2–5 years with mild-to-moderate AD. The randomized, double-blind, vehicle-controlled phase 3 study (NCT04845620) evaluated roflumilast cream 0.05% applied once daily. At week 4, 25.4% of patients achieved vIGA-AD success and secondary endpoints, like EASI-75 and WI-NRS improvement [99].

Roflumilast is FDA-approved in topical formulations for mild to moderate AD treatment (patients ≥6 years), plaque psoriasis, and seborrheic dermatitis [100].

#### 5.3.3. Difamilast

Difamilast (OPA-15406) possesses a 3,4-dialkoxyphenyl group, similar to that of roflumilast, which plays a key role in its binding affinity. Preclinical studies revealed potent anti-inflammatory properties in the context of AD. Difamilast inhibits the production of cytokines such as TNF-α in both human and mouse peripheral blood mononuclear cells. It mitigates skin inflammation in a mouse model of chronic allergic contact dermatitis, its efficacy being higher than that of crisaborole. Furthermore, its topical application resulted in minimal systemic absorption, reducing the risk of adverse effects such as nausea and diarrhea typically associated with systemic PDE4 inhibition. These findings indicated that difamilast had a favorable pharmacological and safety profile for the treatment of inflammatory skin diseases like AD [101].

A phase 2, randomized, double-blind, vehicle-controlled study evaluated the safety and efficacy of difamilast in 73 Japanese pediatric patients with AD, aged 2–14 years. Patients were treated with either difamilast (0.3% or 1%) or a vehicle ointment twice daily for 4 weeks. No serious adverse events were reported, and all treatment-related events were mild-to-moderate. All treatment groups showed significantly greater improvements than the vehicle group in IGA, EASI score, pruritus, and other clinical measures, indicating that difamilast is a safe and effective treatment for pediatric AD [102].

In a phase 3 randomized, double-blind trial, difamilast 1% ointment showed significantly better outcomes than vehicle (IGA score of 0/1 at week 4: 38.46 vs. 12.64%). Difamilast substantially improved the EASI score. Treatment-related adverse events were mild or moderate, difamilast being well-tolerated [103]. Similarly, a meta-analysis of five randomized controlled trials including 1009 patients supported these findings, reporting significant improvements in IGA and EASI scores at week 4. No significant differences in treatment-related adverse events were noted between difamilast and placebo, confirming the treatment’s safety [104].

Difamilast is approved and marketed in Japan since 2021, available in 0.3% and 1% ointment formulations for the treatment of AD in patients aged 2 years and older. It is not approved by the FDA [88].

#### 5.3.4. Apremilast

Apremilast is an oral PDE4 approved in the United States for the treatment of active psoriatic arthritis in adults and of moderate-to-severe plaque psoriasis in patients who are candidates for phototherapy or systemic therapy. Chemically, it contains the pharmacophore 3,4-dialkoxyphenyl group, which is important for binding to the enzyme. Several phase 2 trials assessed the efficacy of apremilast in adult AD. Apremilast significantly reduced the EASI score after 3 months of treatment (at 20 mg: 19%; at 30 mg: 39%). Nausea and diarrhea were the most commonly reported side effects [105].

In a double-blind, placebo-controlled phase 2 trial, the efficacy, safety, and pharmacodynamics of apremilast was assessed in adults with moderate-to-severe AD. Participants were randomized to receive either a placebo, 30 mg of apremilast twice daily (APR30), or 40 mg twice daily (APR40) for 12 weeks. After 12 weeks, all participants received APR30 or APR40 for another 12 weeks. At week 12, among 185 patients, the APR40 group showed significant improvements in the EASI (−31.6%) compared to APR30 (−11.0%) and placebo. The APR40 group also showed a notable reduction in T helper 17/22 markers like IL-17A, IL-22, and S100A7/A8. However, APR40 had more adverse events, including six cases of cellulitis, leading to its discontinuation by a safety monitoring committee. APR30’s safety profile was consistent with known side effects, including nausea, diarrhea, headache, and nasopharyngitis [106].

#### 5.3.5. Orismilast

Orismilast is a potent inhibitor of all PDE4B and PDE4D splice variants, while demonstrating reduced potency toward the PDE4C2 and PDE4A10 subtypes, with IC_50_ values near or below 100 nM. Orismilast contains a 2,6-dichloropyridyl moiety, a structural element also present in roflumilast N-oxide active metabolite [107].

In a 16-week, multicenter, randomized, placebo-controlled Phase IIb trial (Adesos trial, NCT05469464), oral orismilast (20, 30, or 40 mg) was evaluated in adults with moderate-to-severe AD. All doses showed rapid and significant pruritus reduction by week 2. Orismilast was well tolerated, with a safety profile consistent with PDE4 inhibitors, gastrointestinal-related adverse events such as diarrhea and nausea being the most commonly reported [108].

#### 5.3.6. PF-07038124

PF-07038124 features a 1,2-oxaborolan-2-ol ring, a boron-based heterocyclic scaffold structurally analogous to that of crisaborole. Additionally, it incorporates a 3,4-dialkoxyphenyl moiety, a common pharmacophore in the class of PDE inhibitors. It is a selective PDE4B2 inhibitor developed for topical use, based on the soft-drug strategy that ensures rapid metabolic deactivation upon systemic absorption, in order to reduce systemic side effects while maintaining local efficacy in the skin [86].

A multicenter, randomized, double-blind Phase 2a study evaluated the efficacy and safety of PF-07038124 in individuals with mild-to-moderate AD and plaque psoriasis. The trial enrolled 104 participants who were assigned to receive either PF-07038124 0.01% ointment or a matching vehicle, applied once daily for six weeks. By week 6, patients receiving PF-07038124 demonstrated substantially greater reductions in EASI (−74.9%) and Psoriasis Area and Severity Index (PASI) scores (−4.8) compared to those treated with vehicle (−35.5% and 0.1, respectively). The incidence of adverse events was similar across groups, with no local application site reactions reported for PF-07038124, supporting its potential as a well-tolerated and effective topical therapy [109].

#### 5.3.7. Lotamilast

Lotamilast (E6005, RVT-501) is a selective phosphodiesterase-4 (PDE4) inhibitor characterized by a quinazoline core bearing a 3,4-dialkoxyphenyl moiety. This structural motif is integral to its binding within the PDE4 catalytic domain, facilitating high-affinity interactions with the enzyme’s active site [100,110]. It demonstrated significant antipruritic effects in animal models by reducing scratching behaviors. These effects were correlated with the reduction of pro-inflammatory cytokines such as TNF-α, IL-4, and IL-13, resulting in the stabilization of skin immune responses [111]. Lotamilast directly inhibited proteinase-activated receptor 2 (PAR2)-induced pruritus. PAR2 is a key receptor involved in the sensation of itching, particularly in AD. Activation of PAR2 leads to the release of leukotriene B4 (LTB4), a mediator of itching [112]. It effectively inhibits LTB4 production, further contributing to its antipruritic effects and also attenuates C-fiber nerve activity, which is responsible for transmitting itch signals from the skin to the central nervous system. Thus, it interrupts the itch-scratch cycle, providing relief from chronic scratching behaviors that exacerbate skin damage in dermatitis [113].

Human clinical studies have demonstrated the efficacy of lotamilast in treating AD. In a phase 2, randomized, double-blind, vehicle-controlled trial involving Japanese adults with AD, the drug was well-tolerated with no serious adverse events. Over the course of 4 weeks, significant improvements were observed in the EASI, SCORAD, and pruritus scores compared for the treated group compared to the vehicle group. The group that continued the treatment for an additional 8 weeks showed further significant reductions in these scores, supporting its efficacy in managing AD symptoms over longer periods [114]. A 2-week application of the compound in pediatric patients with mild-to-moderate pathology resulted in improved lesion severity and pruritus, further supporting its potential as a safe and effective topical treatment for AD in both adult and pediatric populations [115].

#### 5.3.8. HSK44459

HSK44459 is a selective PDE4B inhibitor developed by Haisco to provide anti-inflammatory and anti-fibrotic effects with fewer side effects than non-selective PDE4 inhibitors. The chemical structure of HSK44459 remains proprietary and has not been disclosed in the public domain. A Phase 2 clinical trial (NCT06996912) is designed to evaluate the effectiveness and safety of HSK44459 in treating adults diagnosed with AD. Conducted across multiple centers, the study uses a randomized, double-blind, placebo-controlled, parallel-group design [116].

## 6. Aryl Hydrocarbon Receptor Modulating Agents

### 6.1. Background

The Aryl Hydrocarbon Receptor (AhR) is a ligand-activated transcription factor that plays a key role in sensing environmental, dietary, microbial, and metabolic signals. It’s part of the basic helix-loop-helix (bHLH) and Per-Arnt-Sim (PAS) family of proteins. The AhR plays a crucial physiological role in modulating the body’s response to environmental toxins and maintaining immune homeostasis [117]. In the skin, AhR activation promotes epidermal differentiation and strengthens barrier integrity by upregulating structural proteins such as FLG, loricrin, and involucrin. Beyond its role in keratinocyte biology, AhR influences innate and adaptive immune responses, modulating cytokine expression and supporting the production of antimicrobial peptides that defend against microbial invasion. In the immune compartment, AhR contributes to the delicate balance between regulatory T cells and effector T cell subsets [118,119].

### 6.2. Mechanism of Action

In AD, the abnormal activation of the AhR is particularly associated with the overproduction of IL-22, a pro-inflammatory cytokine secreted primarily by T-helper 22 cells [120]. Elevated levels of IL-22 contribute to the disruption of skin barrier integrity by impairing keratinocyte differentiation and promoting epidermal thickening, which exacerbates the symptoms of AD. This results in dry, scaly, and inflamed skin, characteristic of the condition [121]. Furthermore, AhR activation impacts the production of other cytokines such as IL-17, a key player in inflammatory responses. IL-17, mainly produced by T-helper 17 cells, is linked to the recruitment of neutrophils and the promotion of inflammation in the skin. In AD, increased IL-17 levels can further disrupt the barrier function by causing keratinocyte hyperproliferation and contributing to chronic inflammation. AhR activation, therefore, amplifies immune dysregulation by affecting these cytokines, leading to worsened skin barrier function [122].

### 6.3. Representatives

#### Tapinarof

Chemically known as 3,5-dihydroxy-4-isopropyl-trans-stilbene, it features a hydroxylated stilbene backbone and exhibits a physicochemical profile defined by moderate molecular weight and pronounced lipophilicity, attributes that confer an efficient dermal penetration. Tapinarof is a bioactive metabolite isolated from the bacterium *Photorhabdus luminescens*. It is known for its anti-inflammatory properties and is the active ingredient in 1% topical formulations, approved as tapinarof in the US and under the name benvitimod in China [123].

A phase 2b double-blind, randomized controlled trial evaluated the efficacy and safety of tapinarof cream in adolescents and adults with AD. The study revealed that 53% of patients using tapinarof 1% twice daily achieved the IGA response (clear or almost clear skin), compared to 24–28% in the vehicle groups. The tapinarof 1% group also had significantly higher rates of achieving and over 75% improvement in EASI and a ≥3-point improvement in pruritus. Common adverse events included folliculitis, headache, and upper respiratory infections, with the tapinarof 1% concentration being more effective than the 0.5% concentration [124]. Tapinarof cream 1% once daily significantly improved efficacy and was well tolerated in patients with moderate-to-severe AD, including children over 2 years old, in the phase 3 ADORING 1 and 2 trials. It significantly improved both symptoms and quality of life in patients with AD [125]. Concerns about the long-term risks of AhR activation, including a potential link to squamous cell carcinoma remain unconfirmed in clinical practice [125].

A multicenter, randomized phase II trial in China assessed the efficacy and safety of 1.0% and 0.5% tapinarof cream, 0.1% tacrolimus, and placebo in adults with AD. Over six weeks, participants applied the treatments twice daily. At week 6, 62.1% of the 1.0% tapinarof group, 31.0% of the 0.5% group, and 58.6% of the tacrolimus group achieved an IGA score of 0 or 1, compared to 39.3% in the placebo group [126]. The compound was well tolerated, with all adverse events being mild and including mostly application site reactions [127].

## 7. Histamine H4 Receptor Antagonists

The histamine H4 receptor (H4R), identified in 2000, is the newest member of the histamine receptor family and plays a key role in immune regulation, particularly in inflammatory and allergic responses. Emerging evidence positions H4R as a promising therapeutic target for AD, alongside other inflammatory conditions like asthma and allergic rhinitis and several H4R antagonists have progressed into clinical trials for these indications. In the skin, H4R is expressed in both the epidermis and dermis, with higher levels in the epidermis, suggesting its involvement in cutaneous immune responses and barrier function. This localization supported the research for drugs to target it [128].

### 7.1. JNJ-39758979

JNJ-39758979, the first H4R antagonist to enter clinical trials, rapidly reduced histamine-induced itching in healthy volunteers, corroborating its role in pruritus. It was then evaluated in a 6-week, phase 2a, randomized, double-blind trial (NCT01497119) involving Japanese adults with moderate AD. The study was terminated early after two participants developed agranulocytosis, likely due to reactive metabolites rather than direct H4R inhibition [129].

### 7.2. Adriforant

Adriforant (ZPL389), an H4R antagonist, was studied in preclinical murine models and a clinical trial for AD. In vitro, it competitively blocked murine H4R, inhibiting ERK phosphorylation, normalizing transcriptional changes in mast cells, and reducing histamine-dependent calcium flux in neurons. In vivo, it suppressed histamine-induced itch and alleviated inflammation in the MC903 dermatitis mouse model. The preclinical benefits did not translate clinically, as the phase 2 trial (NCT03948334) failed to meet efficacy endpoints, highlighting the limits of H4R antagonism alone in AD.

### 7.3. Izuforant

Izuforant (LEO 152020/JW1601), an oral H4 receptor inverse agonist with dual anti-pruritic and anti-inflammatory effects, did not meet the primary endpoint in a phase 2 trial (NCT05117060) for moderate-to-severe AD. The 16-week, triple-blind, placebo-controlled study showed no statistically significant improvement in EASI scores compared to placebo. Following these results, the development program was terminated [130,131].

## 8. Sphingosine-1-Phosphate Receptor Modulators

Sphingosine-1-phosphate receptor (S1PR) agonists act through an initial activation of the receptors, followed by their internalization and downregulation, effectively silencing the S1P signaling cascade. Fingolimod, was approved in 2010 for relapsing-remitting multiple sclerosis and is the only S1PR agonist currently approved for clinical use. The attention has turned to the therapeutic potential of targeting S1PR pathways in various inflammatory skin disorders. Preclinical studies have assessed the effects of topical S1PR agonists in animal models of conditions such as AD, allergic dermatitis, and psoriasis. However, these approaches have also highlighted challenges, notably the risk of systemic absorption and unintended effects beyond the skin [132].

### 8.1. Etrasimod

Etrasimod, a selective oral modulator of S1P receptors 1, 4, and 5, is being investigated for its potential in treating inflammatory skin diseases, including AD. A recent Phase 2 trial tested its efficacy and safety in adults with moderate-to-severe AD. Participants were randomized to receive daily doses of 1 mg or 2 mg or placebo over 12 weeks. The primary endpoint of EASI-75 score reduction did not reach statistical significance, but some efficacy was observed for etrasimod 2 mg on other clinical measures. Safety profiles were acceptable across all groups, with no serious adverse events reported [133]. A phase 2/3 trial testing etrasimod 2 mg daily in adults with treatment-resistant AD was terminated early due to lack of efficacy at interim analysis, with no safety concerns reported [134].

### 8.2. BMS-986166

BMS-986166, a prodrug of the active phosphorylated metabolite BMS-986166-P, presents an improved cardiac safety profile in preclinical studies compared to other S1P1R modulators [135]. In preclinical studies, including a widely cited mouse model of AD, BMS-986166 demonstrated significant anti-inflammatory effects, such as reduced skin lesions and immune cell infiltration. The compound progressed to a Phase 2 clinical trial (NCT05014438) in adults with moderate-to-severe AD. Although the trial was completed, an insufficient number of participants limits its value [67].

### 8.3. SCD-044

SCD-044, also known as vibozilimod, is an oral, selective S1PR1 agonist developed for inflammatory skin conditions like plaque psoriasis and AD [136]. In June 2025, Sun Pharma reported that SCD-044 failed to meet primary efficacy endpoints in two phase 2 trials for plaque psoriasis (NCT04566666) and AD (NCT04684485). Although the drug showed good safety and reduced lymphocyte counts, it did not achieve significant improvements in PASI-75 or EASI-75 at week 16 versus placebo. As a result, the company discontinued its development [137].

## 9. Discussion and Future Directions

Small molecule therapies have undoubtedly expanded the therapeutic options for AD, offering oral and topical alternatives to conventional treatments. However, their mechanistic focus remains narrow, with currently approved agents largely targeting two pathways: JAK inhibition and PDE4 suppression. While the rapid approval of multiple JAK inhibitors underscores the appeal of this strategy, their clinical use has been tempered by significant safety concerns, including venous thromboembolism, major cardiovascular events, and opportunistic infections. These adverse effects raise questions about the suitability of systemic immunomodulation for a primarily cutaneous disease. This is particularly relevant as AD is a chronic, relapsing disease that often necessitates long-term treatment to maintain disease control. Targeting STAT6 is an attractive therapeutic approach because of its pivotal role in IL-4 and IL-13 signaling, both driving the Th2-mediated inflammation. This approach remains in its early stages, making it difficult to fully assess its therapeutic potential, but it could emerge as a promising option for future management of AD.

PDE4 inhibitors have also expanded treatment options for AD, with both topical and oral formulations available. However, their utility is constrained by modest efficacy in moderate-to-severe disease and tolerability issues such as application site pain for topical agents and gastrointestinal side effects for systemic use. These adverse effects are partly due to non-selective inhibition of PDE4 isoforms, particularly PDE4D, which is implicated in emetic and gastrointestinal responses. Future development of PDE4B-selective inhibitors may offer safer, more effective long-term management of this chronic, relapsing disease.

H4R antagonists have long been explored as a potential strategy for AD due to their role in modulating itch and immune cell chemotaxis. However, despite early promise in preclinical models, clinical development has so far failed to yield a successful therapy. Agents such as adriforant showed modest efficacy in clinical trials and were discontinued. Moreover, the recent success of JAK inhibitors and PDE4 inhibitors, which offer broader anti-inflammatory effects and more robust efficacy, has likely shifted research and investment priorities away from H4-targeted therapies. Similarly, S1PR modulation appears to be challenging at present due to the limited efficacy demonstrated by some candidates and the occurrence of systemic adverse effects such as bradycardia and hypertension.

Other intracellular targets are under investigation, including potassium channels such as Kv1.3, which regulate T-cell activation and migration, and chemokine receptors such as CCR4 (C-C chemokine receptor type 4), which mediate Th2 cell trafficking to the skin [138]. RPT193, an oral CCR4 antagonist, was evaluated in a Phase 1a/1b trial for safety, pharmacokinetics, and efficacy in healthy volunteers and patients with moderate-to-severe AD. The drug was well tolerated with no serious adverse events [139].

Currently, small molecule use in AD is empirical, guided by disease severity rather than molecular profiling. To enable precision medicine, validated biomarkers are needed to identify dominant pathogenic pathways in individual patients, along with practical diagnostic tools for routine use. Progress in biomarker discovery and accessible testing will be key to matching small molecules to the right patient at the right time. Topical small molecules are an attractive strategy to limit systemic exposure and associated adverse effects. Agents such as ruxolitinib and roflumilast have demonstrated efficacy in mild-to-moderate AD, though local irritation and insufficient penetration into deeper skin layers remain challenges. One potential approach to address this limitation is the development of prodrugs based on already approved systemic agents. By modifying their physicochemical properties to enhance skin permeation and release the active moiety in situ, such prodrugs could improve local bioavailability while minimizing systemic absorption. Alternatively, soft drugs like leczacitinib offer a promising strategy. Designed to be rapidly inactivated after local action, these compounds could limit systemic exposure while maintaining efficacy in AD.

## Figures and Tables

**Table 1:** Summary of clinical outcome measures in atopic dermatitis trials.

Endpoint	Description	Strengths	Limitations	References
Investigator’s Global Assessment (IGA)	a clinician-rated scale assessing overall atopic dermatitis severity on a 5-point scale: 0 = clear (no inflammatory signs), 1 = almost clear (minimal signs of erythema and infiltration), 2 = mild (mild erythema, infiltration, and possibly minimal oozing), 3 = moderate (distinct erythema, infiltration, oozing/crusting), 4 = severe (severe erythema and infiltration with extensive oozing/crusting and/or lichenification).	○ simple and quick to use ○ widely recognized by regulatory authorities ○ useful as a primary endpoint	○ subject to inter-observer variability ○ less sensitive to partial improvements	[10,11]
Eczema Area and Severity Index (EASI)	○ validated scoring system ○ assesses erythema, induration, excoriation, and lichenification ○ analyzes four body regions (head/neck, upper limbs, trunk, lower limbs) ○ weights regions by body surface area (10%, 20%, 30%, 40%)	○ widely adopted in trials ○ quantitative ○ endorsed by HOME initiative	○ requires training ○ time-intensive ○ excludes subjective symptoms such as itch	[12,13]
SCORAD (Scoring Atopic Dermatitis)	a composite index (0–103) combining three components: (1) Extent of disease—percentage of body surface area involved using the “rule of nines” (head/neck = 9%, each arm = 9%, trunk = 36%, each leg = 18%) (2) Intensity—scoring six clinical signs (erythema, edema/papulation, oozing/crusting, excoriation, lichenification, dryness) each on a scale from 0 (absent) to 3 (severe) (3) Subjective symptoms—patient-reported pruritus and sleep loss, each scored 0–10 on visual analog scale	○ captures both clinician-assessed and patient-reported aspects of AD. ○ widely used in European studies and in pediatric trials. ○ comprehensive for monitoring chronic disease.	○ complex and time-consuming to calculate ○ subjective components may introduce variability ○ less commonly adopted in recent regulatory trials compared to EASI	[14]

**Table 2:** Summary of key Janus kinase (JAK) inhibitors approved for the treatment of atopic dermatitis (AD).

Drug	Mechanism	Approved Dose	Efficacy Endpoints	Adverse Effects
Upadacitinib	JAK1 inhibitor	15 mg or 30 mg oral, once daily	Measure Up 1: EASI-75 achieved by 70% (15 mg) and 80% (30 mg) vs. 16% on placebo Measure Up 2: EASI-75 achieved by 60% (15 mg) and 73% (30 mg) vs. 13% on placebo	very common: acne, upper respiratory infections, nasopharyngitis, headache, elevated CPK—frequently observed across trials common: neutropenia, herpes simplex/zoster, lab changes (CPK, liver enzymes)—dose-dependent but generally mild
Abrocitinib	JAK1 inhibitor	100 mg or 200 mg, oral, once daily	JADE MONO-1, MONO-2, COMPARE: EASI-75 at week 12 achieved in 40–69% of patients with 100 mg	very common: headache, nausea, acne, nasopharyngitis, herpes simplex, elevated CPK, vomiting, dizziness, abdominal pain common: infections, hematologic lab changes, diarrhea, conjunctivitis
Baricitinib	JAK1/JAK2 inhibitor	2 mg or 4 mg, oral, once daily	BREEZE-AD: EASI-75 EASI-75 at week 16 achieved in 13% (1 mg) and 24% (2 mg)	very common: nasopharyngitis common: headache, upper respiratory tract infections, herpes simplex
Ruxolitinib	JAK1/JAK2 Inhibitor	1.5% topical cream, twice daily	TRuE-AD1: EASI-75 at week 8 achieved by 56.0% (0.75%), 62.1% (1.5%) TRuE-AD1: EASI-75 at week 8 achieved by 51.5% (0.75%), 61.8% (1.5%)	common: naso- pharyngitis, upper respiratory tract infection, headache, application site burning, application site pruritus
Delgocitinib	pan-JAK inhibitor	2%, 0.5%, and 0.25% topical formulation	QBA4-1: mEASI-75 at week 4 achieved by 10.9% and at week 24 by 22.7% QBA4-2: mEASI-75 at week 52 achieved by 27.5%	common: nasopharyngitis, contact dermatitis, acne, application site folliculitis

## Abbreviations

The following abbreviations are used in this manuscript:

AD	atopic dermatitis
AhR	aryl Hydrocarbon Receptor
cAMP	cyclic adenosine monophosphate
CPK	creatine phosphokinase
EASI	Eczema Area and Severity Index
EMA	European Medicines Agency
FDA	U.S. Food and Drug Administration
FLG	filaggrin
H4R	histamine H4 receptor
IGA	Investigator’s Global Assessment
IL	interleukin
ITK	interleukin-2-inducible T-cell kinase
JAK	Janus kinase
mAb	monoclonal antibody
PASI	Psoriasis Area and Severity Index
PDE	phosphodiesterase
PROTACs	proteolysis-targeting chimeras
S1PR	Sphingosine-1-phosphate receptor
SCORAD	SCORing Atopic Dermatitis
SH2	Src homology 2
STAT	signal transducers and activators of transcription
TYK2	tyrosine kinase 2
